# Electrochemical and biological characterization of Ti–Nb–Zr–Si alloy for orthopedic applications

**DOI:** 10.1038/s41598-023-29553-5

**Published:** 2023-02-09

**Authors:** Aydin Bordbar-Khiabani, Michael Gasik

**Affiliations:** Department of Chemical and Metallurgical Engineering, School of Chemical Engineering, Aalto University Foundation, 02150 Espoo, Finland

**Keywords:** Biochemistry, Chemistry, Engineering, Materials science

## Abstract

The performance of current biomedical titanium alloys is limited by inflammatory and severe inflammatory conditions after implantation. In this study, a novel Ti–Nb–Zr–Si (TNZS) alloy was developed and compared with commercially pure titanium, and Ti–6Al–4V alloy. Electrochemical parameters of specimens were monitored during 1 h and 12 h immersion in phosphate buffered saline (PBS) as a normal, PBS/hydrogen peroxide (H_2_O_2_) as an inflammatory, and PBS/H_2_O_2_/albumin/lactate as a severe inflammatory media. The results showed an effect of the H_2_O_2_ in inflammatory condition and the synergistic behavior of H_2_O_2_, albumin, and lactate in severe inflammatory condition towards decreasing the corrosion resistance of titanium biomaterials. Electrochemical tests revealed a superior corrosion resistance of the TNZS in all conditions due to the presence of silicide phases. The developed TNZS was tested for subsequent cell culture investigation to understand its biocompatibility nature. It exhibited favorable cell-materials interactions in vitro compared with Ti–6Al–4V. The results suggest that TNZS alloy might be a competitive biomaterial for orthopedic applications.

## Introduction

The demand for artificial implants is growing as the elderly population is growing up in the various countries increases^1^. Approximately 80% of commercial implant devices are made of metallic biomaterials for correcting deformities in the skeletal system^2,3^. Commercially pure titanium (CP-Ti, Grade 2, UNS R50400) is conventionally used as metallic biomaterials in dental devices, joint orthopedic replacements, and cardiovascular stents because of its proper corrosion resistance and satisfactory biocompatibility^4^. CP-Ti does not have all desirable properties for biomedical implants, so Ti–6Al–4V alloy (also known as Grade 5 alloy, UNS R56400) with higher strength is also most widely used^5^. However, there is a growing concern on corrosion and corresponding elution of aluminum and vanadium that have toxic and side effects on long-term applications^6,7^ and newer improved titanium alloys with lower elastic modulus, higher strength, and superior biological performance are being developed^7,8^.

One of the developed alloys without aluminum and vanadium is based on the Ti–Nb–Zr system (UNS R58130 by ASTM F1713) has more biocompatible Nb and Zr elements, and lower Young’s modulus in a range of 60–80 GPa^9–11^. Combining Nb and Zr in this alloy resulted in a near β-Ti phase structure more corrosion resistant than α-Ti and β-Ti phases but it did not have achieved a desired level of biocorrosion resistance without compromising its reactivity to the cells^12^. Among other possible additives silicon has been considered a vital element in the human body for biological reactions, providing a driving force for the growth and development of new bone and connective tissues^13,14^.

After a biomaterial implantation, the body’s immune system is activated to protect the host against infections and tissue damage, additionally complicated with a foreign body reaction^15,16^ and surface deposition of various biomolecules and proteins such as albumin^17^. Leukocytes increase their consumption of oxygen by using the respiratory burst to produce reactive oxygen species (ROS), lactic acid, including hydrogen peroxide (H_2_O_2_) and its derivatives, and hypochlorous acid (HOCl) into the extracellular medium^16,18–20^. For bone tissue, osteoclasts also express HCl which together creates an oxidative acidic environment with pH decrease from neutral to 5 and lower during inflammation^18^. In the severe inflammatory condition, neutrophils, macrophages, and microorganisms can create a very oxidative and acidic medium with pH ⁓ 2–3 surroundings the implant, even sufficient to dissolve very resistant materials such as gold^21^. The passive protective film on titanium in these conditions start to undergo degradation, accelerating corrosion rate and causing surface roughening and formation of intensely hydrated porous TiOOH compound^22–25^. Recent studies showed that the combination of albumin and H_2_O_2_ accelerated the corrosion rate of CP-Ti and Ti–6Al–4V^26,27^ and the presence of lactic acid and H_2_O_2_ decreased the corrosion resistance considerably^28,29^. As a result of the complicated in vivo environment, corrosion of Ti implants can be considerably accelerated, resulting in a significantly shorter implant life and higher risk of failures^30,31^. Therefore, proper alloying additives to high-performance titanium alloys are required to prevent implant failures in inflammatory conditions. Silicon has been introduced to some Ti-based alloys^32–34^, but it has not yet been sufficiently analyzed when added to the Ti–Nb–Zr system. So, there is an interest to assess as Ti–Nb–Zr–Si alloy for potential material for orthopaedic applications.

In this work, a novel Ti–Nb–Zr–Si (TNZS) alloy was made and its in vitro characteristics including the corrosion performance and biological response to cell cultures were evaluated. Severe inflammatory conditions for electrochemical corrosion characterization were simulated by incorporating of H_2_O_2_, bovine serum albumin (BSA), HCl and calcium l-lactate hydrate (CLH) into the normal physiological solution (corrosion behavior of titanium was previously studied when albumin and lactic acid are added to H_2_O_2_-containing solutions separately^26–29,35–40^, but not yet when both are added simultaneously). To the best of authors’ knowledge, the in vitro corrosion of CP-Ti, Ti–6Al–4V, and especially TNZS has never been reported in the severe inflammatory conditions. The electrochemical corrosion measurements of TNZS were conducted in the inflammatory and severe inflammatory conditions simulated by the addition of H_2_O_2_, and H_2_O_2_/BSA/CLH to the acidified phosphate-buffered saline (PBS), respectively. These in vitro characteristics of TNZS alloy as potential orthopedic biomaterials are discussed and compared with conventionally used CP-Ti and Ti–6Al–4Valloys.

## Results and discussion

### Microstructure and thermodynamic calculations

**Figure 1:**
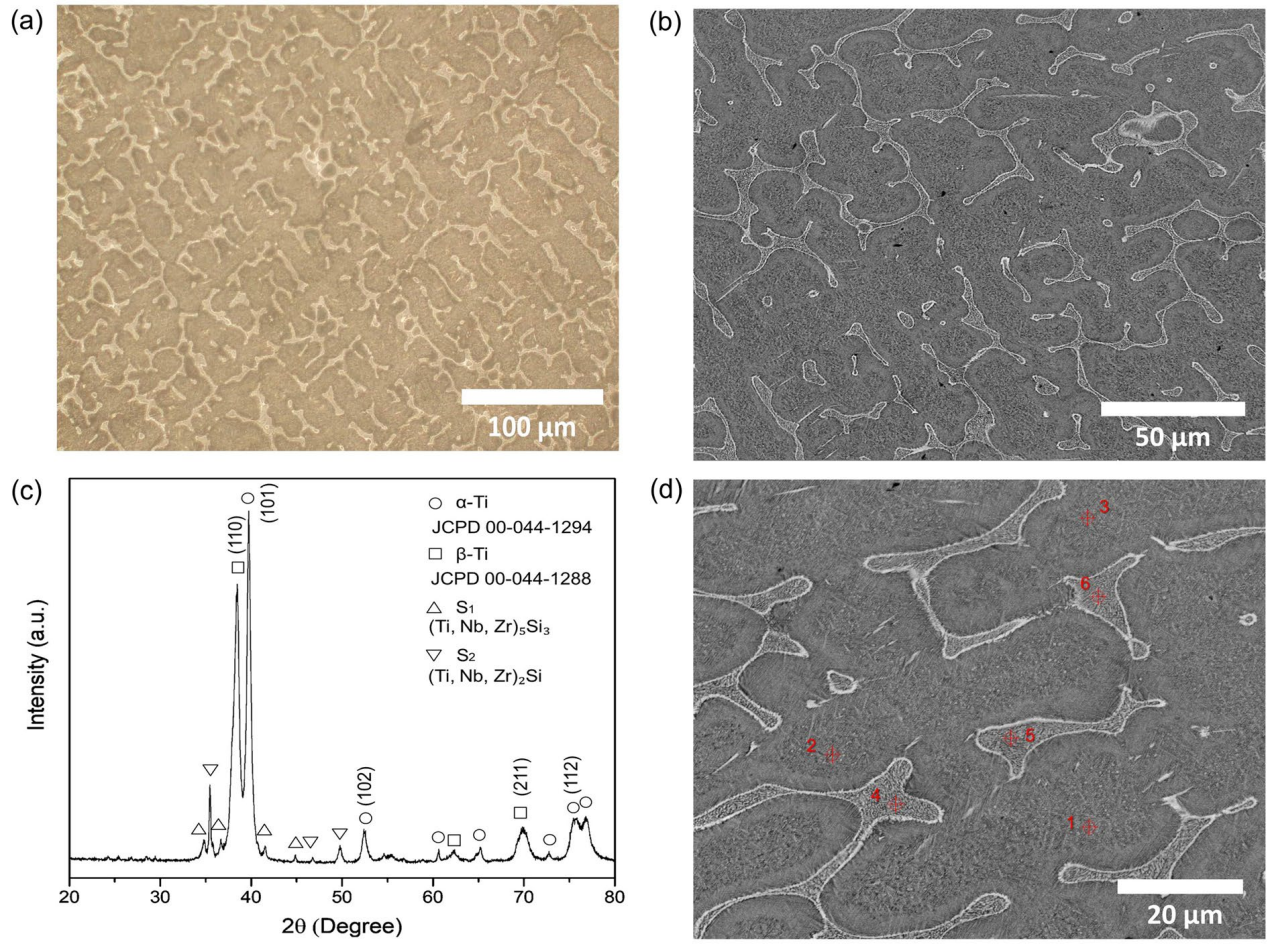
(**a**) OM image, (**b**) back-scattered (BSE) SEM micrograph, (**c**) XRD pattern, and (**d**) magnified BSE-SEM micrograph with EDS point analyses of TNZS alloy.

The optical microscopy (Fig. 1a) and SEM (Fig. 1b) images of TNZS alloy clearly show consisting lamellar eutectics discontinuously distributed on the matrix. Figure 1c shows a typical XRD pattern of the TNZS alloy at α-Ti, β-Ti, and intermetallic silicide (S_1_ of the M_5_Si_3_ type and S_2_ of M_2_Si type) phases were identified and indexed well as marked at the diffractograms. The EDS analysis was carried out on six points on TNZS microstructure at the different regions in the precipitated phase and matrix to examine the elemental distribution as presented in supplementary Table ST1 (Supplementary Materials data file), exhibiting a clear elements partition between these phases. The metallic matrix has ratio Nb:Zr = 2:1 whereas in the silicides it is the opposite Nb:Zr = 1:2, indicating that addition of silicon to Ti–Nb–Zr alloy and subsequent formation of silicides leads to higher binding of zirconium in the intermetallic phases leaving niobium to enrich metallic phase^34,41^.

Thermodynamic calculations results show that (Supplementary Fig. S1a) during solidification primary ß-phase is forming first following precipitation of M_5_Si_3_ silicide (S_1_) as the major intermetallic phase and subsequent α-ß transus of the metallic solid solution phase. The M_2_Si (S_2_) phase can only form at local areas where zirconium concentration is higher (Supplementary Fig. S1b), as this phase does not form in the binary Ti–Si or Nb–Si systems. Enrichment of metallic phase with niobium might be seen beneficial from the point of corrosion resistance of this alloy. One can also expect that if the phase transformations are following the diagram (Supplementary Fig. S1a) than S_1_ silicides will precipitate in the ß-rich matrix providing additional strengthening against deformation. This requires an additional analysis as the mechanical properties of the TNZS alloy are not considered in this study, but it indicates a possibility of extra options for the alloy properties modification via heat treatment.

### Electrochemical characterization

#### Open circuit potential

**Figure 2:**
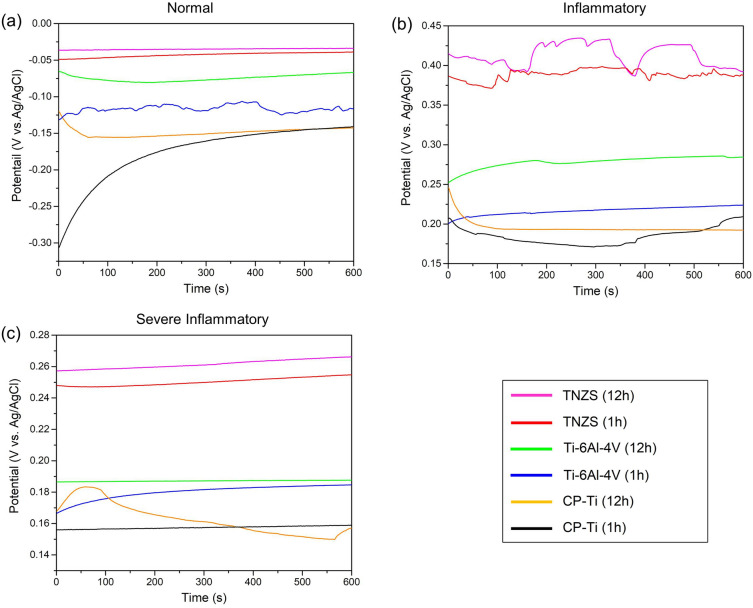
OCP monitoring of the CP-Ti, Ti–6Al–4V, and TNZS specimens exposed in (**a**) simulated normal, (**b**) inflammatory, and (**c**) severe inflammatory conditions for 1 h and 12 h.

Figure 2 shows the OCP evolution of specimens after 1 and 12 h of immersion in normal, inflammatory, and severe inflammatory conditions media. The OCP values increased with immersion time in all conditions except CP-Ti sample in the severe inflammatory condition. This indicates that oxide layers have thickened or reconstructed in terms of their structure or chemical composition, which can be the result of the suppression of anodic reactions and a decrease in the anodic current density^42^. Under all conditions, TNZS alloy has shown a nobler potential compared with CP-Ti and Ti–6Al–4V, presumably due to formation of a passive layer consisting of more niobium oxide, also denser and defect-less than oxide layers usually forming on CP-Ti and Ti–6Al–4V^43,44^. Observed fluctuations in the OCP of the TNZS alloy specimens in the inflammatory environment can be caused by the high resistance of the passive layer, which may become broken and then to repassivated in the presence of hydrogen peroxide.

As seen in Fig. 2a,b, when H_2_O_2_ is added to PBS (to simulate the inflammatory conditions), the OCP values shift to more electropositive values. This can be explained by H_2_O_2_ decomposition to H_2_ and O_2_, acting as an extra cathodic reaction to the oxygen reduction^43^. Moreover, Tengvall’s studies suggest that the positive OCP in H_2_O_2_-containing solutions are often caused by intermediates with a strong oxidizing character adsorbing to the passive film on Ti, such as HO_2_ and HO^44,45^. In contrast, in a severe inflammatory solution containing BSA and CLH in addition to H_2_O_2_ in PBS (Fig. 2c), the OCP of all specimens was consistently lower than that in the presence of PBS and H_2_O_2_ alone (inflammatory), but higher than in PBS alone (normal media). Recent studies suggested that BSA adsorbs on titanium surfaces by electrostatic interactions or chemisorption through amino/carboxylate groups, decreasing the rate of cathodic reduction of H_2_O_2_^46–48^. The same behavior has been reported in the addition of the CLH to the H_2_O_2_-containing solutions, which significantly blocks the cathodic site by adsorption on the surface, resulting in lower values of the OCP^28,29^. This does however not mean that the BSA and CLH inhibit the corrosion of Ti in the severe inflammatory medium and it seems necessary to perform PDP and EIS tests for further electrochemical studies.

#### Electrochemical impedance spectroscopy

Figure S2a–i in the Supplementary Data file represent the Nyquist, Bode modulus and Bode phase plots of EIS tests, respectively. The symbols are the experimental data and the solid lines are fitting data. The equivalent circuit (Supplementary Fig. S3) based on a single time constant on Bode plots was selected to represent the electrochemical corrosion phenomenon in normal, inflammatory and severe inflammatory condition, which contains the solution’s resistance (R_s_), constant phase element (CPE), and passive layer’s resistance (R_pl_)^49^. Some heterogeneities, such as surface roughness, impurities, or defects, made it necessary to introduce a CPE element instead of a simple capacitor^48^. The CPE element impedance (Ohm·cm^-2^) can be described by Q and n parameters through following equation^49^:

1 $${\text{Z}}_{\text{CPE}}=\frac{1}{\text{Q}{(\text{j}\upomega)}^{\text{n}}},$$

where *n* is the power coefficient varied between 0 and 1, ω is an angular frequency, and $j= \sqrt{-1}$. Interfacial capacitance is usually approximated by the value of Q when *n* → 1, but this approach has been found to be inaccurate^49^. The following Eq. (2) is used to calculate the effective capacitance (C_eff_) values of interfacial capacitance so that a more realistic value can be assigned^50^, which has been shown more accurate:

2 $${\text{C}}_{\text{eff}}=\sqrt[\text{n}]{\text{Q}\cdot {\text{R}}_{\text{pl}}^{1-\text{n}}}.$$

A semiconductive oxide film thickness value (d_eff_) formed on titanium alloys can be evaluated using this equation^51^ as:

3 $${\text{d}}_{\text{eff}}=\frac{\upvarepsilon {\upvarepsilon }_{0}}{{\text{C}}_{\text{eff}}},$$

where ε is the layer’s dielectric constant (taken as 45 for pure Ti and its alloys^52^), and ε_0_ is the vacuum permittivity (8.85 × 10^−14^ F·cm^−1^). The fitted and calculated values through Eqs. (1)–(3) are listed in Supplementary Table ST2. The R_pl_ values of all specimens increased after 12 h in all solutions, suggesting the formation and growth of the passive protective film. In inflammatory media the concentration of H_2_O_2_ is decreasing with time due to the decomposition of H_2_O_2_ to H_2_O and O_2_^43,53^ and here the reduction of the media oxidative power facilitates the self-healing of the passive layer. It can be seen from Supplementary Table ST2 that the largest R_pl_ values among the specimens were obtained for the TNZS alloy.

When comparing the resistances of passive layers using the designated R_pl_ values, the inflammatory condition has a destructive effect on the layers and caused a decrease in passive layers’ resistance. This is due to the formation of Ti(IV)-H_2_O_2_ complex compounds in response to the reaction between Ti and H_2_O_2_^54^. The corrosion resistance in the severe inflammatory conditions has noticeably deteriorated because of the co-presence of BSA, CLH, and H_2_O_2_ where R_pl_ values show a synergetic effect on both BSA and CLH on increase of the degradation of titanium alloys. It was claimed that the high oxidation potential of ROS intermediates in the presence of biomolecules produces a strong electric field across the passive layer, leading to an acceleration in the oxidation process of Ti^55,56^. Another possible explanation for decreased R_pl_ in severe inflammatory condition is due formation of a lactate-chelating compound when titanium reacts with CLH^57,58^. Similar behavior in Ti was observed when exposed to lactic acid in artificial saliva^57^.

Bode phase plots (Fig. S2i) show that TNZS alloy immersed in the severe inflammatory media exhibited phase angles close to 80°, confirming its excellent corrosion resistance and slower reactivity compared to other specimens with phase angles of 50°. This indicates that the microstructure and chemical composition determines the level of protection, governed by the passive oxide layers formed on TNZS. Calculated C_eff_ values are within a range of 10^–5^ F·cm^−2^ (Supplementary Table ST2), which is typical of what has been reported for bulk Ti and its alloys in simulated body fluids^59,60^. Calculated values of passive layer thickness (d_eff_) did not exceed a few nanometers, which is comparable to values reported by other electrochemical and non-electrochemical studies on Ti surfaces^61^.

#### Potentiodynamic polarization

**Figure 3:**
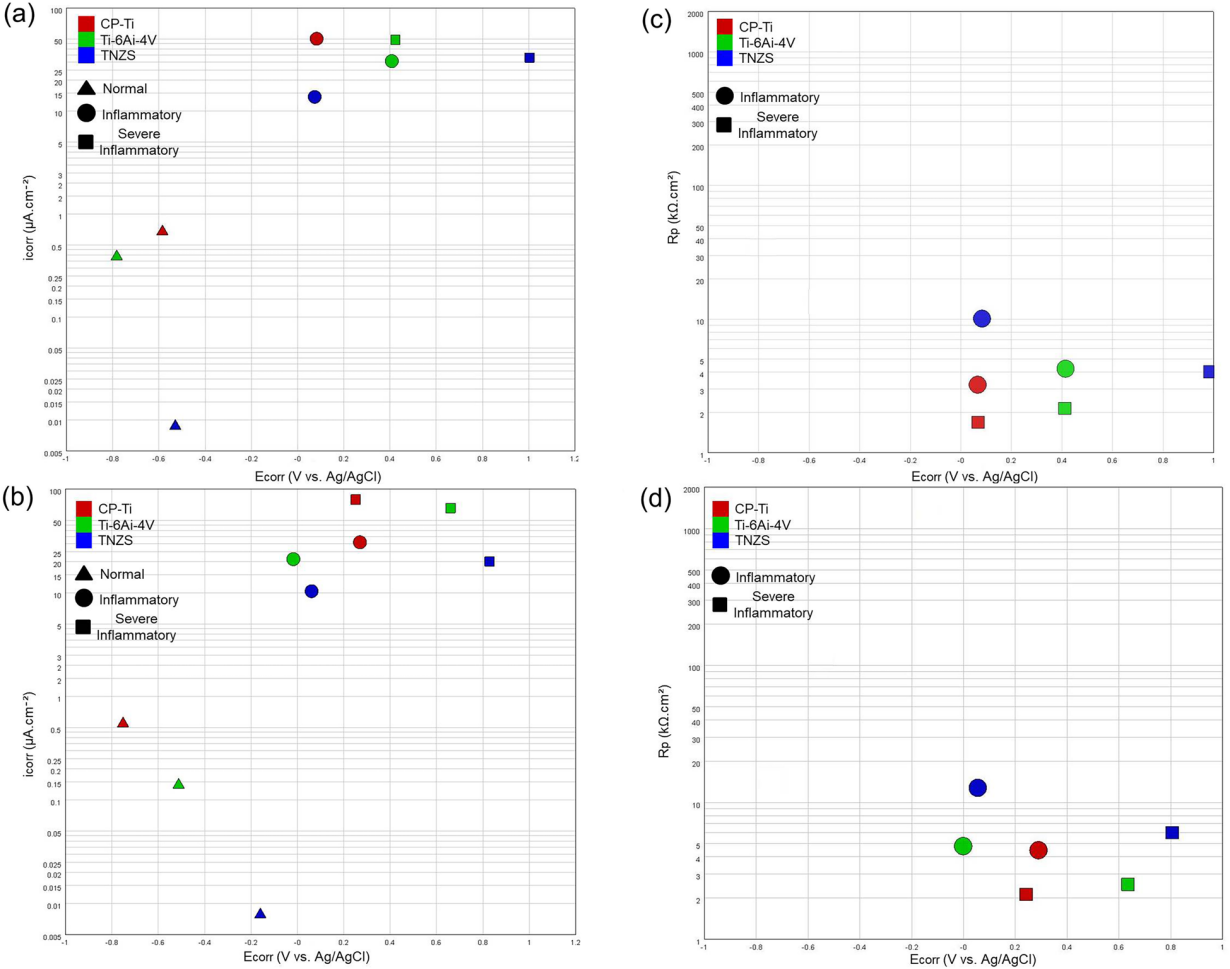
i_corr_ vs. E_corr_: (**a**) 1 h and (**b**) 12 h; R_p_ vs. E_corr_: (**c**) 1 h and (**d**) 12 h.

Supplementary Figure S4 illustrates PDP curves recorded after 1 and 12 h immersion in normal, inflammatory and severe inflammatory conditions for all materials. Here the corrosion behavior of specimens is being evaluated by the corrosion potential (E_corr_), corrosion current density (i_corr_), and polarization resistance (R_p_). The latter could be calculated by the Stern–Geary Equation^38^:

4 $${\text{R}}_{\text{p}}=\frac{{\upbeta }_{\text{a}}{\upbeta }_{\text{c}}}{2.3{\text{i}}_{\text{corr}}({\upbeta }_{\text{a}}{+\upbeta }_{\text{c}})},$$

where β_a_ and β_c_ are respectively Tafel slopes of anodic and cathodic branches on the PDP curve. The E_corr_, i_corr_, and β_a_ and β_c_ values were determined by the Tafel extrapolation method and reported in Supplementary Table ST3. The typical passivation behavior of Ti-based materials may be responsible for the absence of Tafel behavior in anodic branches under normal conditions, thus making it impossible to determine the β_a_, and only β_c_ values are reported^62^. The plotted i_corr_ vs. E_corr_, and R_p_ vs. E_corr_ are shown in Fig. 3.

It is seen that in all conditions i_corr_ of specimens has decreased after 12 h immersion. For the normal media this can be associated with the formation of a stable surface oxide film protecting surfaces from further attack. The increased corrosion resistance in inflammatory and severe inflammatory conditions can be attributed to the catalytic decomposition of H_2_O_2_ to H_2_O and O_2_ during the 12 h immersions decrease the oxidizing power of the solutions^43,53^. From anodic branches of PDP curves it is seen that specimens had passive behavior in the normal media but not in the inflammatory and severe inflammatory conditions^23,27,29^. As seen in Fig. 3a,b, addition of H_2_O_2_ to the PBS has lead to increase of i_corr_ values for all specimens, indicating a higher corrosion rate in the inflammatory conditions. These results are consistent with previous studies of increased i_corr_ and decreased R_p_ of CP-Ti and Ti–6Al–4V in the H_2_O_2_-containing biological solutions. Similar to the OCP shown in Fig. 2b, the E_corr_ values were electropositive for the inflammatory condition (H_2_O_2_-containing PBS) compared to the normal condition. In the presence of BSA, CLH and H_2_O_2_ in PBS (severe inflammatory media), i_corr_ increases (Fig. 3a,b) and R_p_ decreases (Fig. 3c,d) drastically, suggesting that BSA and CLH along with H_2_O_2_ have a synergetic effect on the corrosion behavior of Ti implants. Some fluctuations can be seen on the branches of PDP curves in Fig. S4 in severe inflammatory condition are attributed to the effect of the adsorbed biomolecules on the titanium surface on anodic and cathodic reactions in this condition. Similar fluctuations in the PDP curves were previously observed in the corrosion studies of stainless steel in bacteria-containing solutions^63^.

For CP-Ti material a gradual increase in i_corr_ was observed in inflammatory and severe inflammatory conditions, suggesting the CP-Ti surface becomes oxidized in the presence of H_2_O_2_, but the formed oxide is dissolved faster than needed to provide proper protection^64^. Ti–6Al–4V alloy showed better corrosion performance than CP-Ti in all conditions which is consistent with the literature^65^. There the lowest corrosion resistance was observed in inflammatory media compared with the normal condition. It has been suggested that the increase in the i_corr_ in this condition is caused by the dissolution of vanadium oxide on the surface of Ti–6Al–4V alloy, and then the generation of vacancies in the oxide layers, further enhanced by H_2_O_2_ and Cl^-^ ions^46,65^. In terms of i_corr_, TNZS alloy showed the lowest values between all specimens in all media and immersion times, and it also had less fluctuations than for Ti–6Al–4V alloy. It was discussed that more fluctuations of current observed in the anodic branches are related to the surface roughening and continuous formation/destruction of metastable pits on the electrode surface^54,66^, indicating that the passive layers on Ti–6Al–4V are less stable in this medium.

The highest corrosion resistance of TNZS can be mainly attributed to the addition of especially Nb, and to lesser extent to Zr and Si elements in the alloy, resulting in the formation of a more inert and stable oxide film. Other studies showed that a ternary Ti–Nb–Zr alloy has improved stability of the surface films exhibiting exceptionally high resistance to oxidizing acids^39,43^. Some reports showed the role of Si in the stabilizing the crystallized phases in Si–Nb-based alloys that might be helpful to promote corrosion resistance^67–70^. It has been also reported that the addition of Si can improve the corrosion resistance of Ti alloys in acidic solutions due to the formation of stable SiO2 and Si-doped TiO2 passive layers on the Ti–Si alloy^34,71^. The impressive corrosion performance of TNZS alloy in inflammatory and severe inflammatory conditions is due to the presence of Nb-enriched α + β matrix and S_1_/S_2_ intermetallic phases. Supplementary Table ST4 compares the corrosion parameters extracted from PDP curves of the TNZS alloy fabricated in this study with Ti–Nb–Zr–Me (Me = metal) alloys reported in the literature, showing that TNZS alloy is less susceptible to corrosion than CP-Ti, Ti–6Al–4 V, and Ti–Nb–Zr–Me^72–76^ alloys when exposed to similar conditions.

#### Surface morphology after PDP tests

**Figure 4:**
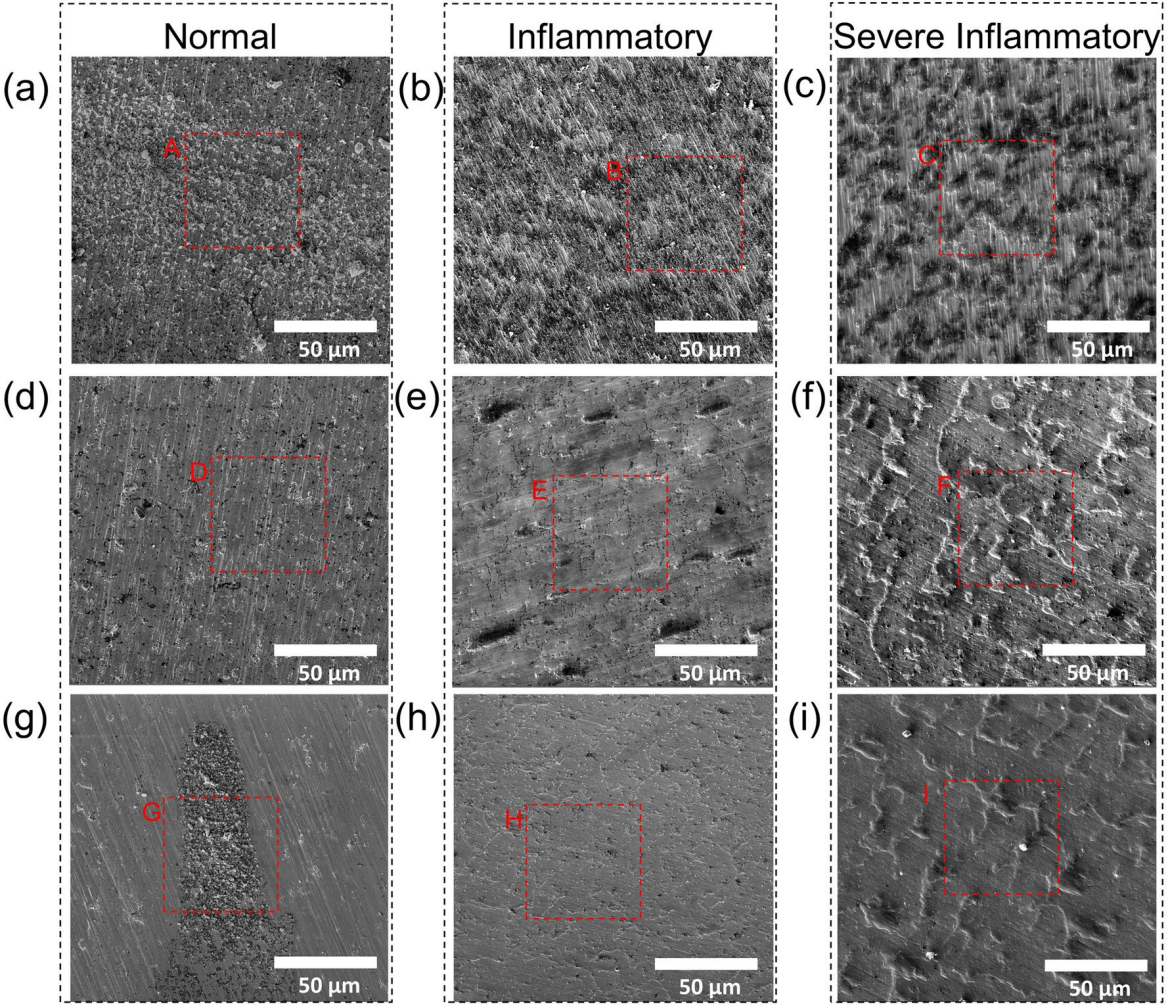
Top-surface SEM micrographs of specimens exposed in simulated normal, inflammatory and severe inflammatory conditions for 12 h: (**a–c**) CP-Ti, (**d–f**) Ti–6Al–4V, and (**g–i**) TNZS.

Figure 4 represents the top-view SEM images of specimens after 12 h immersion and recording the corresponding PDP curves in each simulated solution. The EDS elemental analysis results of specified areas in Fig. 4 are listed in Supplementary Table ST5. There are obvious differences in the surface morphologies between CP-Ti, Ti–6Al–4V, and TNZS specimens in normal, inflammatory, and severe inflammatory conditions. As seen in Fig. 4a–c, the inferior pitting corrosion and surface roughening occurred for CP-Ti, in all simulated conditions. The decrease in the amount of Ti and the increase in the amount of oxygen in inflammatory and severe inflammatory conditions indicate the promotion of the implant’s degradation rate and growth of the oxide layer over time for these environments compared with the normal condition.

In the micrographs of the TNZS alloy exposed in the normal solution (Fig. 4g), there are fewer pits and localized corrosion products, and the polished surface with scratches can still be seen. Figure 4e shows the mud-crack morphology and large pits with an increase in Cl content on the surface of Ti–6Al–4V, suggesting a higher corrosion rate in acidic H_2_O_2_-containing PBS solution. The same morphologies were reported for prolonged exposure to Ti–6Al–4V in inflammatory conditions^23^. The adsorption of Cl^-^ ions to oxygen vacancies in the presence of H_2_O_2_ on the surface of the oxide/solution induces a flux of cation vacancies to the metal oxide interface and their annihilation^77,78^. As shown in Table ST5 for regions C, F, and I, more carbon is present correlating with expected BSA and CLH adsorption on Ti surfaces when immersed in the severe inflammatory condition media^29,79^. These morphological changes in the different conditions are correlated with the variation of *n* values in Eq. (1), as their reduction in inflammatory and severe inflammatory conditions (Table ST2) is indicative of larger surface heterogeneity in these conditions (Fig. 4).

It is evident from Fig. 4f that intergranular voids formed through the dissolution of some material are leading to the degradation of the Ti–6Al–4V surface in the severe inflammatory conditions. It is known that the pitting resistance of Ti alloys can be significantly reduced by the addition of Al and V elements and several studies have reported corrosion of Ti–6Al–4V alloy in the presence of H_2_O_2_ due to vanadium oxide dissolution^77^. Compared with CP-Ti and Ti–6Al–4V materials TNZS showed lower localized corrosion damage (Fig. 4h,i) in inflammatory and severe inflammatory conditions, but small pits and some corrosion products nevertheless have appeared on the surface. The silicide phases can also play a crucial role in the corrosion behavior of the TNZS in acidic H_2_O_2_-containing solutions as recent studies shown e.g. Ti_5_Si_3_ (equivalent to S_1_ phase in the present study) had excellent corrosion resistance in acidified H_2_O_2_ solution^80^. Hence it can be concluded that the impressive corrosion performance of TNZS alloy in inflammatory and severe inflammatory conditions is also enhanced by the presence of silicide intermetallic phases.

### Cells responses

**Figure 5:**
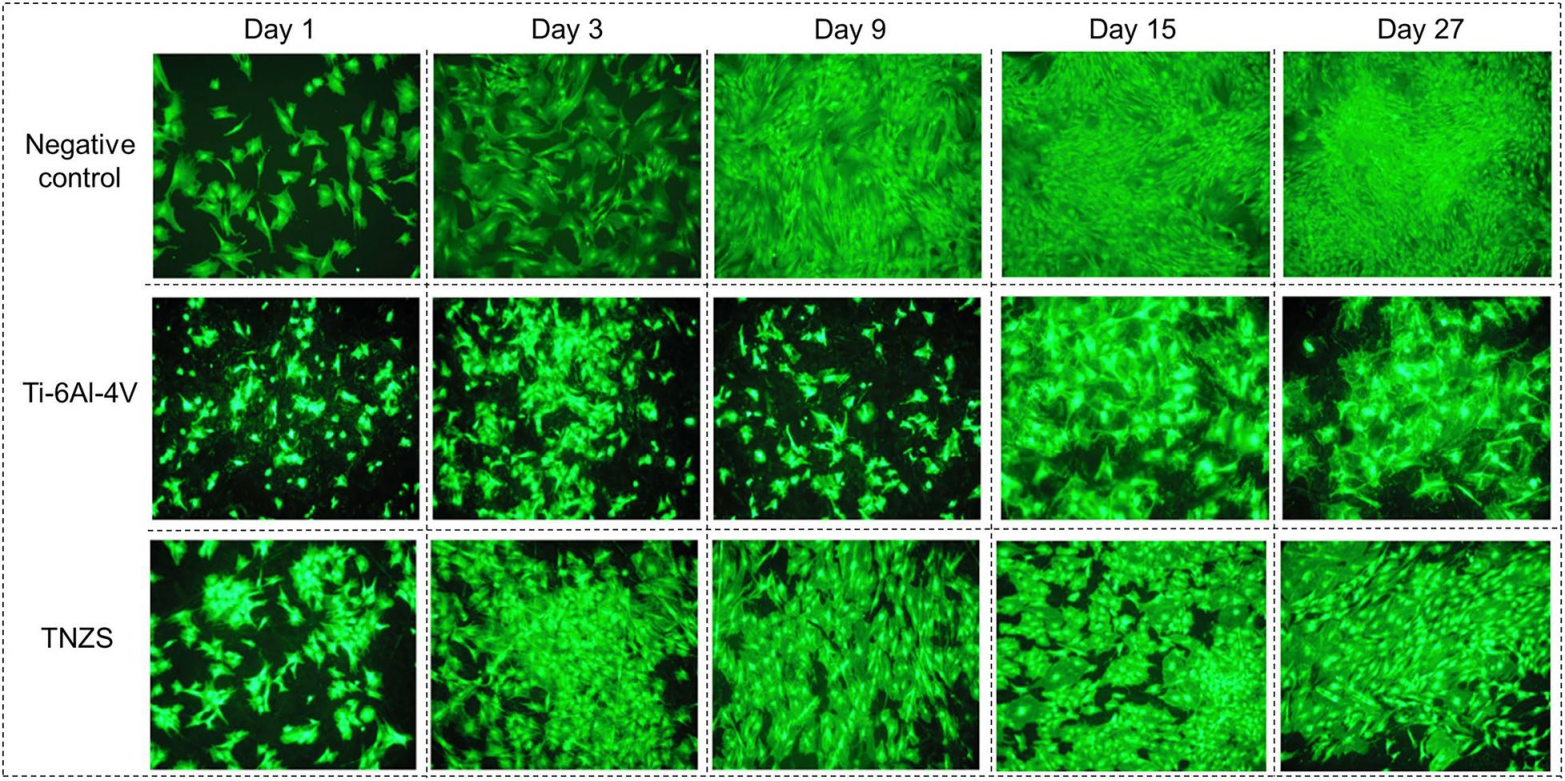
HOC proliferation and growth (× 100) of negative control, Ti–6Al–4V, and TNZS at 1, 3, 9, 15, and 27 days.

The results of the cells-materials interactions are shown in Fig. 5 and Supplementary Figs. S5–S7. For HOC (Fig. 5) it can be visually seen that cells on TNZS are doing much better than on Ti–6Al–4V alloy (with the same surface morphology) although lower than at the negative control. Nevertheless, the HOC coverage area (%) on TNZS remains high enough at 40–45% for days 9 to 27 vs. negative control (40–50%) and especially Ti–6Al–4V (15–25% only). For HUVEC (Fig. S5) this difference is clearly favorite for TNZS (20–25% in day 15) vs. negative control (10%) and Ti–6Al–4V (< 2% only).

In gene expression analysis for HOC, much higher levels of ALP and ColI are seen for TNZS, and these differences are stable in time (Fig. S6a,b). At the first day, Cbfa is well expressed on TNZS indicating potentially high osteogenic nature of the alloy (Fig. S6d), and OC starts to be readable at negative control after day 9 (Fig. S6c), according to differentiation of osteogenic cells. For unknown reasons, OC has not been detected on TNZS alloy, so additional tests are eventually required. For HUVEC (Fig. S7) CD31 and vWf expressions are clear on negative control, eventually decreasing in time as when there are too much cells in vitro, one can observe a decrease of cell activity. Both these factors are however more important for angiogenesis rather than for osteogenesis.

Overall, it could be seen that both TNZS and Ti–6Al–4V alloys have exhibited a good HOC colonization on the substrate, whereas for the latter proliferation rate is more modest when looking to negative control. For HUVEC a little growth was seen on Ti–6Al–4V but much more substantial growth on TNZS. Major osteogenic markers were equivalent or higher on TNZS compared to negative control, which shows it to be a positive candidate for orthopedic applications.

## Conclusions

In summary, this study highlights the electrochemical corrosion and biological behaviors of a novel TNZS alloy for orthopedic applications. The electrochemical measurements studied the effects of immersion time and simulated biological media on the corrosion performance of TNZS, Ti–6Al–4V, and CP-Ti biomaterials. Invasive orthopedic implantation provokes inflammatory and severe inflammatory responses that produce acidic environments with H_2_O_2_ and biomolecules substances near the metallic biomaterial. The inflammatory and severe inflammatory media were mimicked by additions of H_2_O_2_ and H_2_O_2_, BSA, and CLH to the PBS solution, respectively. OCP test showed that the values increased with immersion time for all specimens and TNZS alloy had a nobler potential compared others.

The TNZS alloy showed the highest corrosion resistance in all media and electrochemical testing time, based on EIS and PDP tests’ results. The high corrosion resistance was attributed to the presence of silicide phases in the microstructure of TNZS alloy, which have high stability in acidic environments containing H_2_O_2_. SEM/EDS analyses after the PDP test proved the synergistic effects of BSA and CLH on the corrosion response of titanium biomaterials in severe inflammatory conditions. The TNZS alloy also exhibits excellent biological activity in terms of HOC and HUVEC proliferation and growth, which makes it a promising biomedical material for orthopedic applications.

## Materials and methods

### Sample preparation

The TNZS alloy was prepared from Ti, Nb, Zr, and Si raw materials (Goodfellow Cambridge Ltd., Huntington, UK) with vacuum arc-melting in copper molds, chill cast into steel mold making a rod-like specimen of 20 mm diameter and approximately 100 mm in length. The rod was further wire-cut into specimens without further heat treatment. The starting point of the alloy composition was based on Zr/Nb weight ratios closer to unity, similarly to commercial biomedical alloy TNZ (UNS R58103 by ASTM F1713), with addition of up to 2 wt% Si. The rationale for silicon content was to trigger formation of fine silicides but preserving major metallic matrix, as was calculated with thermodynamic equilibrium as shown below. The chemical composition of the TNZS alloy was determined by inductively coupled plasma optical emission spectrometry (ICP-OES) as 11.15 ± 0.19 wt %Zr, 9.61 ± 0.32 wt %Nb, 1.23 ± 0.20 wt %Si, and Ti as the balance. Commercial purity titanium (CP-Ti, 99.6% purity) and Ti–6Al–4V (Al = 5.5–6.76% wt, V = 3.5–4.5% wt, and Ti = balance) alloy were obtained from Goodfellow Cambridge Ltd. (Huntingdon, UK). Samples were cut into discs 0.5 mm in height and 15 mm in diameter. The surface was ground with 240–2500 grit SiC sandpapers, polished with alumina suspension (0.3 μm, Allied, Canada), then ultrasonically washed with 70% ethanol (Merck, Germany) and deionized water.

### Microstructural characterizations

The microstructures of the polished TNZS alloy were observed through an optical microscope (OM, Leica DM2500, Germany), and a scanning electron microscope (SEM, TESCAN Mira 3, The Czech Republic) equipped with an energy dispersive spectrometer (EDS, QUANTA-450FEI, USA) after chemical etching with Kroll’s reagent (1 ml HF + 2 ml HNO_3_ + 100 ml H_2_O) at room temperature for 5 min. The phase composition of the polished TNZS alloy was identified using X-ray diffractometer (XRD, Philips PW 3040/60, The Netherlands) operated at 30 mA and 40 kV by a monochromatic Cu*K*_α_ as a radiation source with 2θ ranging from 20° to 80°.

### Thermodynamic calculations

Thermodynamic analysis of the TNZS alloy has been made with CALPHAD method^81^. For the solution phases, liquid, b-phase (BCC structure) and a-phase (HCP structure) substitutional solution model (Ti,Nb,Zr,Si) was used to describe their Gibbs energy functions according to the Redlich–Kister formalism^81^. Binary and ternary interaction parameters are obtained from the corresponding sub-systems from own thermodynamic database using FACTSage software (FACTSage 8.0, GTT, Germany). For the silicide phases two sublattice models in the format of (Ti, Nb, Zr)_*a*_Si_*b*_ were applied and in the first sublattice of this model (metal-rich), binary interaction parameters among the elements are estimated by using solubility data in ternary systems but without the ternary parameter. Although the descriptions of the three silicon-contained ternary subsystems have still uncertainties and require further investigations, recent analysis is resulting in more reliable data in the metal-rich side as the ternary Ti–Nb–Zr system has been sufficiently well described^82^. In this work thermodynamic description was deployed only to estimate the expected phases within the composition range of the TNZS alloy.

### Electrochemical measurements

Electrochemical measurements were performed with potentiostat (IviumStat.h standard, The Netherlands) in a conventional three-electrode system with a graphite rod as the counter electrode, Ag/AgCl as a reference electrode (R.E.), and Ti-based specimens as a working electrode (W.E.) with exposed area of 1 cm^2^ (Fig. S8 in the Supplementary Data file). The five phosphate-buffered saline tablets (PBS, Sigma-Aldrich, USA) were dissolved in 1 l deionized water to prepare the basic solution that refers to the normal physiological condition. One tablet dissolved in 200 ml of deionized water yields PBS solution (0.137 M sodium chloride, 0.0027 M potassium chloride, and 0.01 M phosphate buffer), with pH 7.34 at 25 °C. Reagents H_2_O_2_ (30%, Merck, Germany) and hydrochloric acid (HCl; 37%, Merck, Germany) were added to the PBS-based solution to simulate inflammatory conditions. For the severe inflammatory condition H_2_O_2_, HCl, bovine serum albumin (BSA, Sigma-Aldrich, Germany) and calcium l-lactate hydrate (CLH, Sigma-Aldrich, Germany) were all added to the PBS-based solution (Supplementary Table ST6). The pH values and conductivity of the solutions were measured using a Metrohm 691 pH meter, and Mettler Toledo Inlab 730 probe, respectively. The measurements were carried out at 37 ± 0.5 °C and under slight anaerobic conditions by purging air + 5% CO_2_ gas through the solution.

All specimens were immersed in simulated solutions before measurements for 1 and 12 h to evaluate the time-dependent corrosion behavior and formed passive layers stability. The specimens’ open circuit potential (OCP) was recorded during 600 s following each immersion time. Potentiodynamic polarization (PDP) measure–ments were done with a sweep range from − 1.5 to 1.5 V versus OCP voltage at a constant sweep rate of 1 mV·s^−1^. The electrochemical impedance spectroscopy (EIS) was performed in the frequency range of 100 kHz–0.010 Hz under AC voltage of 10 mV in amplitude around OCP potential. EIS data were examined by the ZView (version 3.1, Scribner Associates) software. The data fitting quality was confirmed by chi-squared (χ^2^) values that are in the order of 10^−3^. The reproducibility and reliability of the obtained results have been guaranteed by conducting all tests in triplicate. As a post-corrosion study, the top-view morphology of surfaces exposed to simulated solutions for 12 h was characterized using SEM/EDS after PDP tests.

### In vitro biological characterizations

Biological reactions of the new alloy in comparison with standard titanium alloy Grade 5 (Ti–6Al–4V) were analyzed with human cells (HOC) arising from bone marrow (6th passage) and human endothelial cells (HUVEC; 9th passage, PromoCell) at an independent laboratory for analysis of implantable materials (Laboratoire d’Evaluation des Matériels Implantables—LEMI, Martillac, France) with the details of the protocol as presented earlier^83^. Briefly, study of the HOC and HUVEC adhesion was analyzed with Live/Dead viability kit where live cells are distinguished by the presence of ubiquitous intracellular esterase activity, determined by the enzymatic conversion of the non-fluorescent cell-permeable calcein AM to the intensely fluorescent calcein (green). Nuclei of dead cells are stained by passive transport of ethidium bromide (red).

Cells proliferation was analyzed (LEMI, Matrillac, France) by seeding cells at 10 K (HOC) or 20 K cells·cm^−2^ (HUVEC) in triplicate and counted using a hemocytometer after various incubation times (1, 3, 9, 15 and 27 days at 37 °C in a humidified atmosphere containing 5% vol CO_2_). Samples were covered up first with complete culture medium and incubated at 37 °C during 3 × 30 min to allow serum proteins to absorb on the surface first to mimic in vivo situation following implantation (adsorbed protein layer). Cell counting was performed at the end of the incubation period by detaching cells with using 0.2% wt trypsin in Hank’s balanced Ca/Mg-free solution incubated (2 min) with 0.2% wt Trypan blue in 0.15 M NaCl and counting dead (blue) and living cells (uncolored) with a hemocytometer. Plastic standard PS culture wells were used everywhere as a negative control and Ti–6Al–4V alloy specimens as a comparison material. The analysis of cytoskeleton and focal adhesion formation after 24 h incubation was made through actin, tubulin, vinculin and integrin αvβ3 study with the coating/substrate using immunofluorescence with monoclonals conjugated to fluorescein isothiocyanate isomer I (FITC) or crystalline tetramethyl rhodamine isothiocyanate (TRITC) for vinculin. A nuclear labelling was performed using Hoescht staining^83^. Study of HOC and HUVEC differentiation was carried out at 1, 3, 9, 15 and 27 days of incubation times with cells seeding at 100 K cells·cm^−2^. Total RNA was isolated from the cells after 1 and 3 days of contact, cDNA was synthesized and PCR is performed to analyze the genes with a semi-quantitative method, normalizing results to a housekeeping gene (β-actine). Specific genes were related to HOC (collagen type 1 (ColI), alkaline phosphatase (ALP), osteocalcine (OC), Cbfa (core-binding factor subunit alpha-1, also known as Runt-related transcription factor 2 (RUNX2) which is a protein key transcription factor associated with osteoblast differentiation), and for HUVEC (CD31—a platelet endothelial cell adhesion molecule (PECAM-1) and von Willebrand factor (vWf)), with further protocol details as reported previously^83^.

Image analysis of the results was made with ImageJ (v. 1.53s, NIH, USA) applied on fluorescent images after color thresholding (Huang method in HSV space with green color as the target) to exclude dark background and integrated particle analysis macro; additionally the coverage of the area by the living cells was compared to controls.

## Supplementary Information

Supplementary Information.

## Data Availability

The data used to support the findings of this study are available from the corresponding author upon request.
