# Supplementary material for: Electrochemical and biological characterization of Ti–Nb–Zr–Si alloy for orthopedic applications
Source: Sci Rep. 2023 Feb 9;13:2312. doi: 10.1038/s41598-023-29553-5 (PMC9911760; doi:10.1038/s41598-023-29553-5)
Supplement: Supplementary file 1 — Supplementary Information. [file 41598_2023_29553_MOESM1_ESM.docx]

**Supplementary information**

**Electrochemical and biological characterization of Ti-Nb-Zr-Si alloy for orthopedic applications**

Aydin Bordbar-Khiabani^*^, Michael Gasik

Department of Chemical and Metallurgical Engineering, School of Chemical Engineering, Aalto University Foundation, 02150 Espoo, Finland

*Corresponding author's email: [aydin.bordbarkhiabani@aalto.fi](mailto:aydin.bordbarkhiabani@aalto.fi)


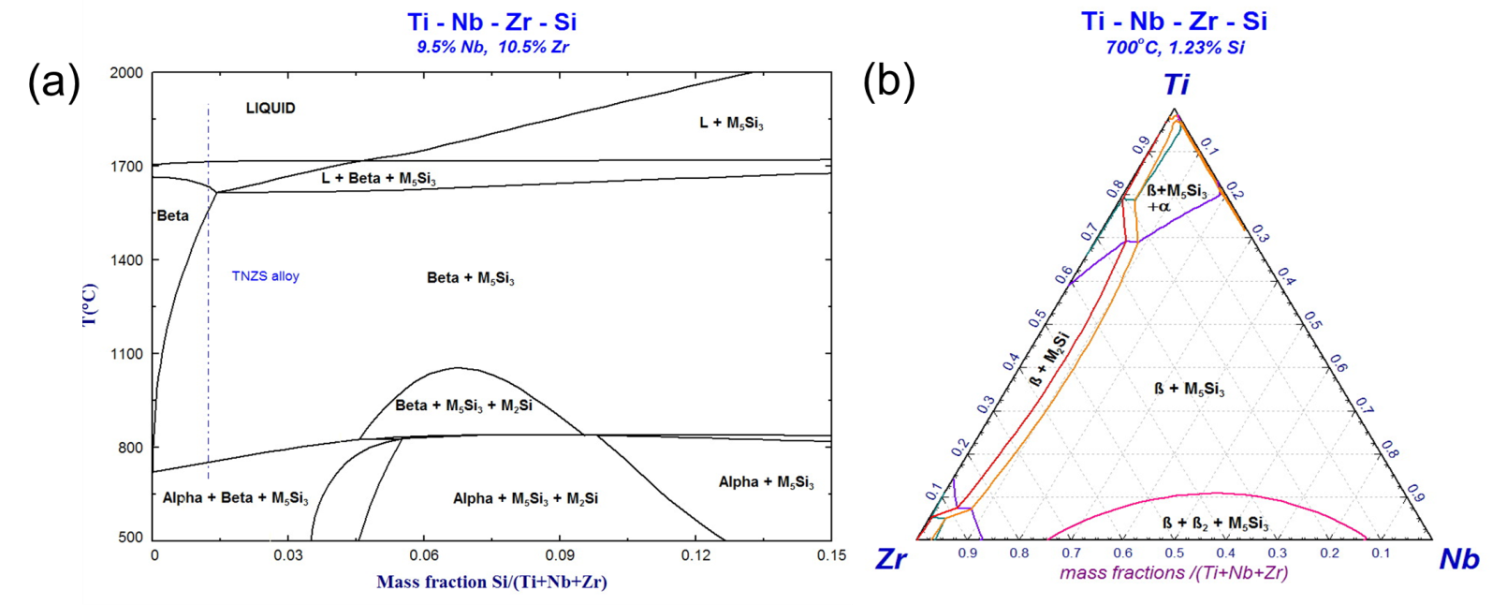


Figure S1. (a) Calculated section of the Ti-Nb-Zr-Si phase diagram for the TNZS alloy composition range and (b) isothermal cross-section at 700°C of the Ti-Nb-Zr-Si system at 1.23% wt. Si.


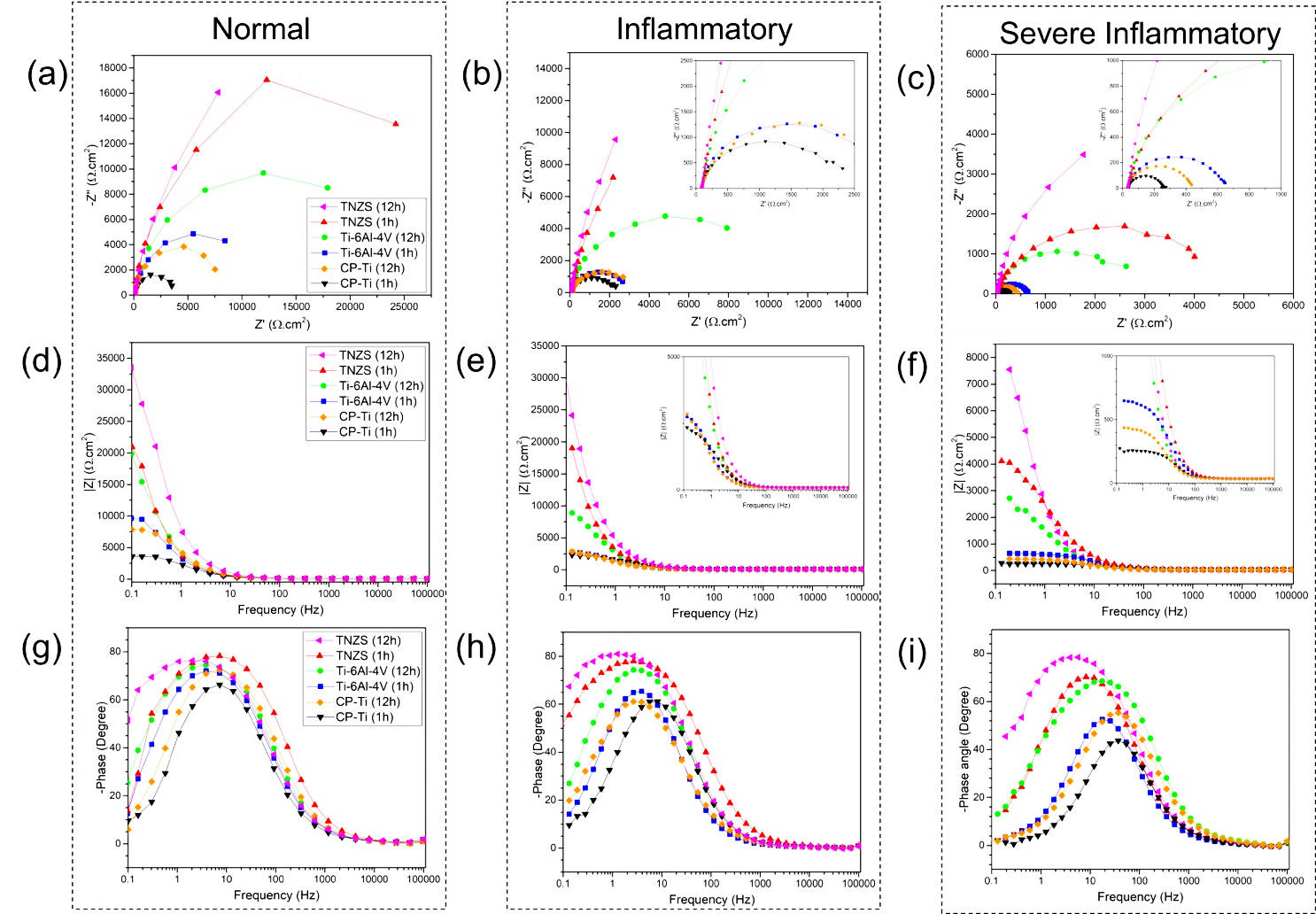


Figure S2. EIS plots of the CP-Ti, Ti-6Al-4V, and TNZS specimens exposed in simulated normal, inflammatory and severe inflammatory conditions for 1 and 12 h: (a-c) Nyquist, (d-f) Bode modulus, and (g-i) Bode phase plots. Some parts of plots have been enlarged for better display.


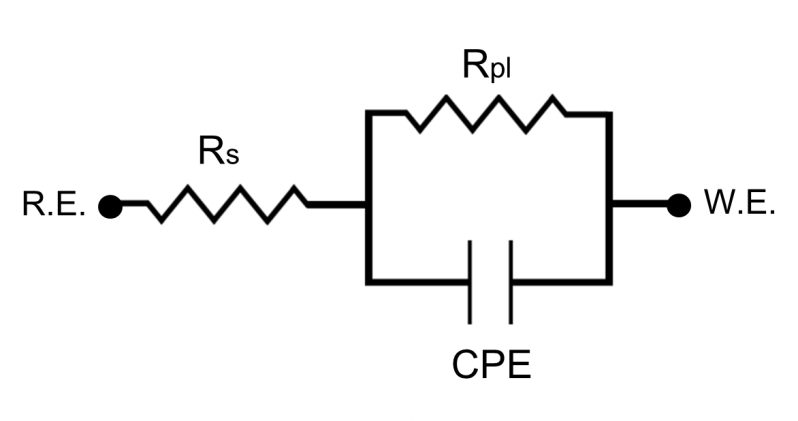


Figure S3. The equivalent circuit employed to fit the EIS data in Figure S3.


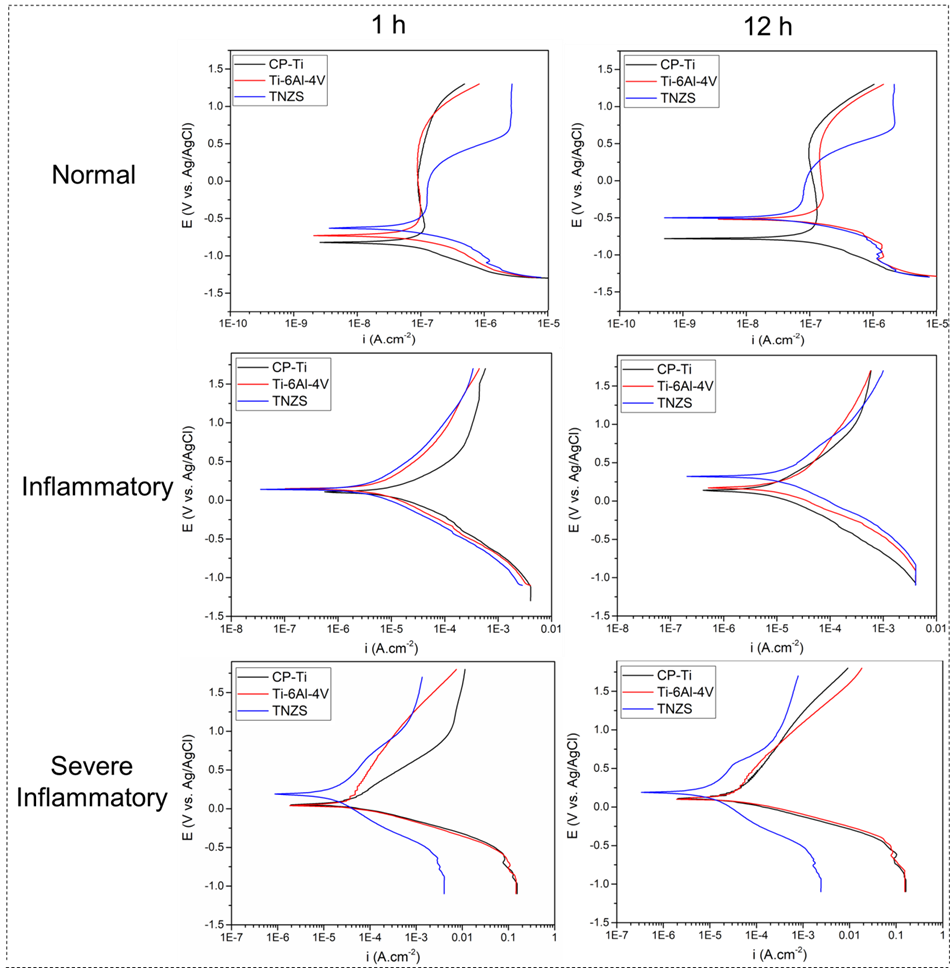


Figure S4. PDP curves of the CP-Ti, Ti-6Al-4V, and TNZS specimens exposed in simulated normal, inflammatory and severe inflammatory conditions for 1 and 12 h.


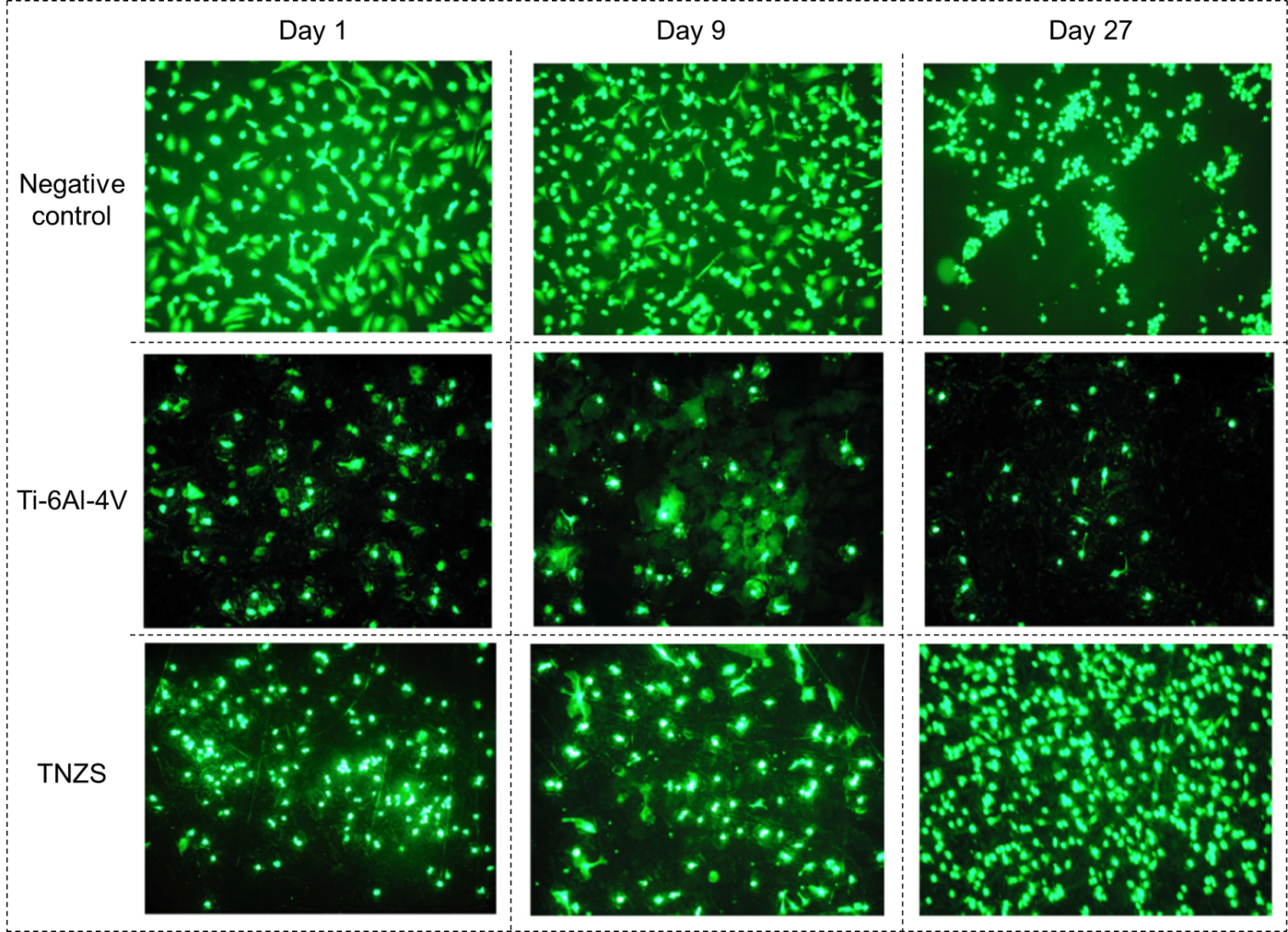


Figure S5. HUVEC proliferation and growth (x100) of negative control, Ti-6Al-4V, and TNZS at 1, 9, and 27 days.


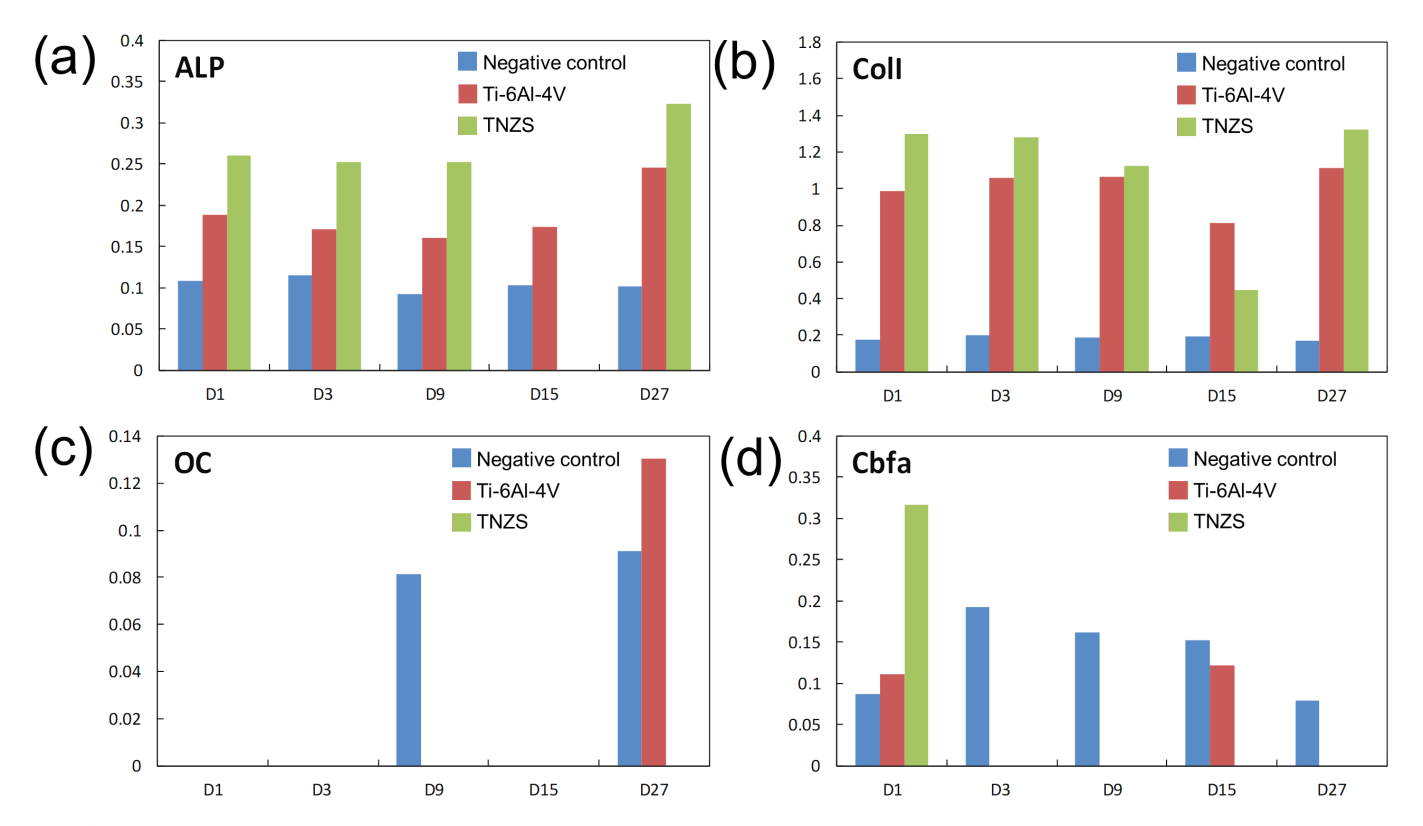


Figure S6. Gene expression analysis of the alloys after day 1, 3, 9, 15 and 27. ALP = alkaline phosphatase, ColI = collagen I, OC = osteocalcine, Cbfa - core-binding factor subunit alpha-1 (RUNX2)


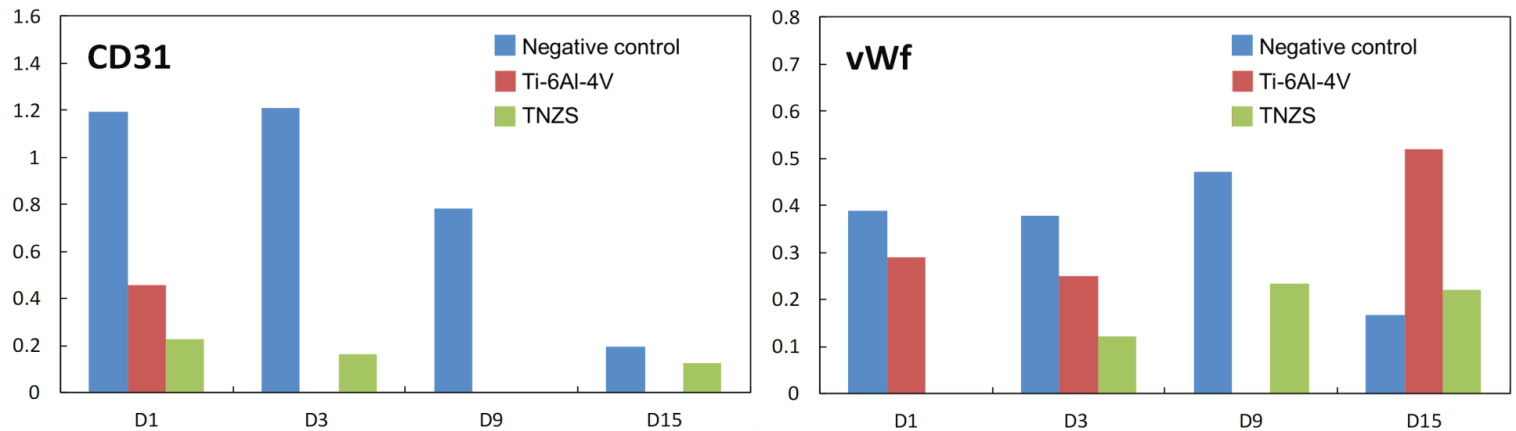


Figure S7. Gene expression analysis for HUVEC after day 1, 3, 9 and 15. CD31 = a platelet endothelial cell adhesion molecule (PECAM-1), vWf = von Willebrand Factor.


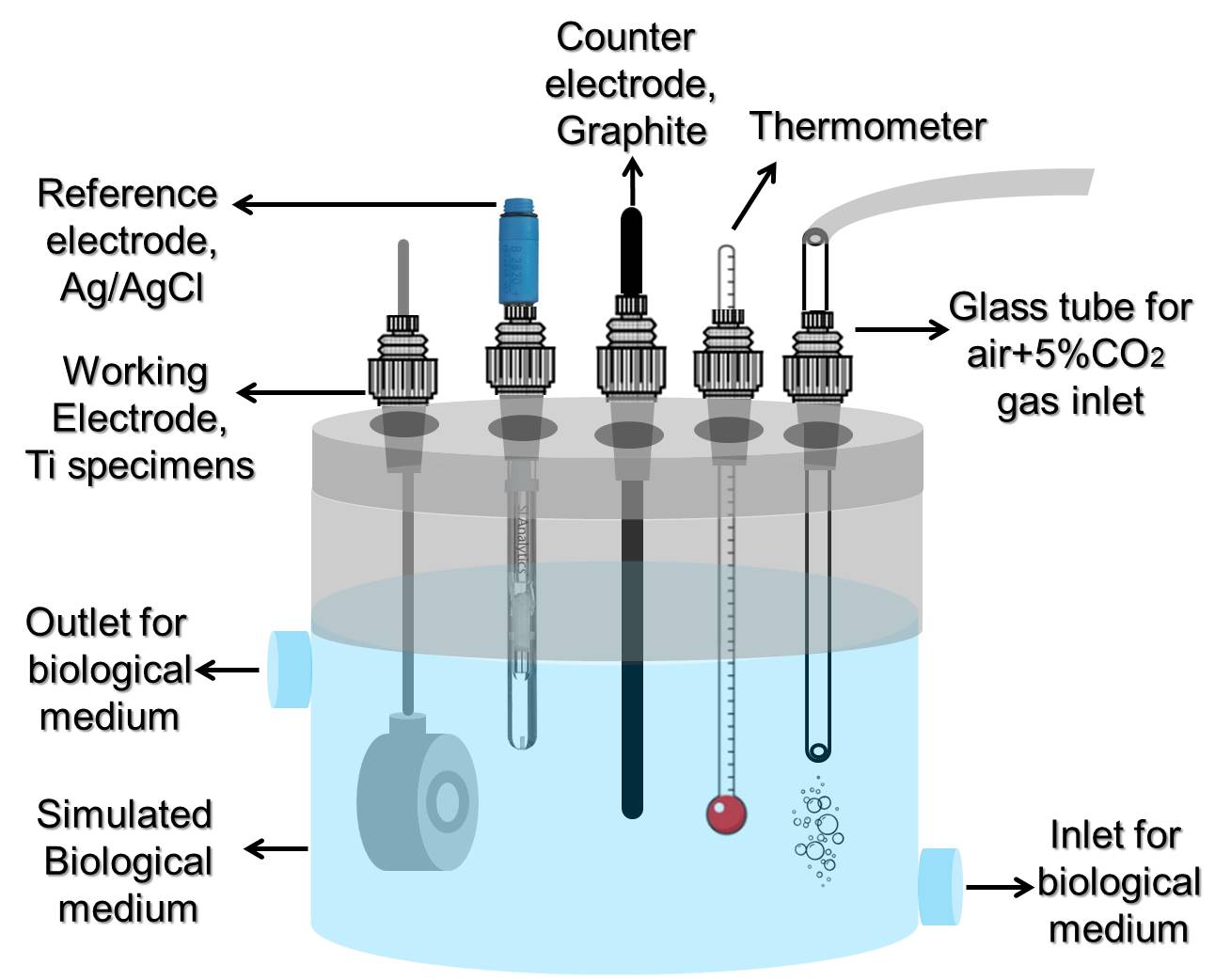


Figure S8. Schematic representation of the electrochemical cell used for in vitro corrosion measurements.

Supplementary Table ST1. Elemental composition of the marked points indicated in Figure 1d by EDS.

| Point No. | Ti | | Nb | | Zr | | Si | |
| --- | --- | --- | --- | --- | --- | --- | --- | --- |
|  | Wt.% | At.% | Wt.% | At.% | Wt.% | At.% | Wt.% | At.% |
| 1 | 73.03 | 82.99 | 17.21 | 10.08 | 8.93 | 5.33 | 0.82 | 1.6 |
| 2 | 72.56 | 82.53 | 17.22 | 10.01 | 9.28 | 5.54 | 0.94 | 1.82 |
| 3 | 74.32 | 84.34 | 16.9 | 9.89 | 8.37 | 4.99 | 0.4 | 0.78 |
| 4 | 62.2 | 69.57 | 11.82 | 6.82 | 19.65 | 11.54 | 6.33 | 12.07 |
| 5 | 63.29 | 70.33 | 10.82 | 6.2 | 19.51 | 11.39 | 6.38 | 12.09 |
| 6 | 61.93 | 68.69 | 9.9 | 5.66 | 21.12 | 12.3 | 7.05 | 13.35 |

Supplementary Table ST2. Parameters determined from fitting of the EIS plots of the CP-Ti, Ti-6Al-4V, and TNZS in different simulated conditions.

| Simulated conditions | Immersion time (h) | Specimens | R_pl_  (kΩ·cm^2^) | Q  (10^-5^Ω^-1^·cm^-2^·S^n^) | n | C_eff_  (10^-5^F·cm^-2^) | d_eff_  (nm) |
| --- | --- | --- | --- | --- | --- | --- | --- |
| Normal | 1 | CP-Ti | 18.23±0.34 | 3.08±0.18 | 0.97 | 3.25±0.16 | 1.22±0.20 |
|  | 1 | Ti-6Al-4V | 36.94±0.28 | 2.88±0.09 | 0.96 | 3.18±0.22 | 1.25±0.24 |
|  | 1 | TNZS | 1453.9±9.43 | 2.90±0.16 | 0.94 | 2.96±0.10 | 1.34±0.19 |
|  | 12 | CP-Ti | 22.17±0.23 | 3.32±0.22 | 0.96 | 3.54±0.43 | 1.12±0.36 |
|  | 12 | Ti-6Al-4V | 121.6±0.12 | 3.04±0.24 | 0.95 | 3.12±0.36 | 1.27±0.22 |
|  | 12 | TNZS | 1715.5±7.31 | 2.76±0.11 | 0.97 | 2.88±0.11 | 1.38±0.09 |
| Inflammatory | 1 | CP-Ti | 7.34±0.22 | 2.41±0.25 | 0.94 | 2.45±0.17 | 1.62±0.16 |
|  | 1 | Ti-6Al-4V | 9.55±1.01 | 2.20±0.18 | 0.96 | 2.38±0.25 | 1.67±0.20 |
|  | 1 | TNZS | 14.01±0.54 | 2.14±0.15 | 0.95 | 2.17±0.34 | 1.83±0.18 |
|  | 12 | CP-Ti | 8.69±0.53 | 2.16±0.19 | 0.96 | 2.19±0.23 | 1.81±0.41 |
|  | 12 | Ti-6Al-4V | 10.30±0.18 | 1.89±0.24 | 0.94 | 2.06±0.43 | 1.93±0.34 |
|  | 12 | TNZS | 17.89±0.26 | 1.85±0.32 | 0.96 | 2.04±0.26 | 1.95±0.12 |
| Severe inflammatory | 1 | CP-Ti | 3.11±0.44 | 1.84±0.17 | 0.92 | 2.05±0.25 | 1.94±0.11 |
|  | 1 | Ti-6Al-4V | 6.27±0.32 | 2.01±0.11 | 0.90 | 2.10±0.41 | 1.89±0.17 |
|  | 1 | TNZS | 10.93±0.10 | 1.92±0.14 | 0.91 | 1.92±0.39 | 2.07±0.10 |
|  | 12 | CP-Ti | 3.76±0.27 | 1.83±0.29 | 0.89 | 1.88±0.33 | 2.12±0.43 |
|  | 12 | Ti-6Al-4V | 8.05±0.25 | 1.59±0.16 | 0.90 | 1.85±0.28 | 2.15±0.15 |
|  | 12 | TNZS | 14.13±0.19 | 1.55±0.21 | 0.91 | 1.76±0.29 | 2.26±0.13 |

Supplementary Table ST3. PDP parameters of the CP-Ti, Ti-6Al-4V, and TNZS in different simulated conditions.

| Simulated conditions | Immersion time (h) | Specimens | E_corr_  (V vs. Ag/AgCl) | β_a_  (V·dec^-1^) | -β_c_  (V·dec^-1^) | i_corr_  (μA·cm^-2^) | R_p_  (kΩ·cm^2^) |
| --- | --- | --- | --- | --- | --- | --- | --- |
| Normal | 1 | CP-Ti | -0.561±0.123 | - | 0.520±0.106 | 0.86±0.20 | - |
|  | 1 | Ti-6Al-4V | -0.787±0.234 | - | 0.443±0.085 | 0.35±0.23 | - |
|  | 1 | TNZS | -0.528±0.345 | - | 0.874±0.154 | 0.010±0.005 | - |
|  | 12 | CP-Ti | -0.745±0.546 | - | 0.359±0.054 | 0.66±0.12 | - |
|  | 12 | Ti-6Al-4V | -0.562±0.105 | - | 0.487±0.123 | 0.12±0.04 | - |
|  | 12 | TNZS | -0.119±0.085 | - | 0.685±0.186 | 0.0085±0.0004 | - |
| Inflammatory | 1 | CP-Ti | 0.055±0.013 | 0.685±0.254 | 0.494±0.213 | 48.09±4.33 | 3.15±0.27 |
|  | 1 | Ti-6Al-4V | 0.420±0.174 | 0.722±0.262 | 0.557±0.211 | 31.54±2.45 | 4.33±0.34 |
|  | 1 | TNZS | 0.092±0.047 | 0.879±0.072 | 0.422±0.112 | 12.76±1.69 | 9.71±1.27 |
|  | 12 | CP-Ti | 0.305±0.138 | 0.996±0.162 | 0.533±0.233 | 32.63±5.36 | 4.62±0.54 |
|  | 12 | Ti-6Al-4V | 0.012±0.0059 | 0.969±0.145 | 0.435±0.135 | 24.91±1.21 | 5.23±0.34 |
|  | 12 | TNZS | 0.051±0.0090 | 0.781±0.157 | 0.421±0.056 | 10.17±2.10 | 12.65±1.56 |
| Severe inflammatory | 1 | CP-Ti | 0.089±0.0217 | 0.957±0.206 | 0.444±0.327 | 85.15±2.19 | 1.54±0.36 |
|  | 1 | Ti-6Al-4V | 0.402±0.135 | 0.881±0.154 | 0.552±0.458 | 61.12±1.16 | 2.41±0.27 |
|  | 1 | TNZS | 0.971±0.270 | 0.529±0.253 | 0.741±0.126 | 33.18±0.45 | 4.04±0.55 |
|  | 12 | CP-Ti | 0.235±0.195 | 0.622±0.116 | 0.444±0.156 | 70.10±2.33 | 1.99±0.40 |
|  | 12 | Ti-6Al-4V | 0.616±0.424 | 0.729±0.298 | 0.453±0.226 | 53.77±1.68 | 2.26±0.37 |
|  | 12 | TNZS | 0.790±0.183 | 0.775±0.177 | 0.575±0.035 | 22.48±1.35 | 6.38±0.86 |

Supplementary Table ST4. The comparison of reported corrosion results of near β-, and β-Ti alloys with the obtained results from TNZS alloy.

| Ti alloys | Immersion time (h) | Corrosive media | R.E. | E_corr_  (V) | i_corr_  (nA·cm^-2^) | R_p_  (kΩ·cm^2^) | Ref. |
| --- | --- | --- | --- | --- | --- | --- | --- |
| Ti-Nb-Zr-Mn | 1 | Ringer’s solution | SCE* | -0.301 | 14.61 | - | [72] |
| Ti-Nb-Zr-Mo | 4 | SBF** | SCE | -0.344 | 71.9 | 829.2 | [73] |
| Ti-Nb-Zr-Ta | 1 | SBF | SCE | -0.454 | 31.01 | - | [74] |
| Ti-Nb-Zr-Mg | 3 | PBS | SCE | -0.614 | 26 | 72.4 | [75] |
| Ti-Nb-Zr-Sn | 1 | SBF | SCE | -0.313 | 12 | 123 | [76] |
| Ti-Nb-Zr-Si | 1 | PBS | Ag/AgCl | -0.528 | 10.24 | - | This study |
|  | 12 |  |  | 0.119 | 8.51 | - |  |

*Saturated calomel electrode

**Simulated body fluid

Supplementary Table ST5. The chemical composition (EDS; wt. %) of the specified regions indicated in Fig. 4.

| Regions | Ti | Al | V | Nb | Zr | Si | O | C | Cl |
| --- | --- | --- | --- | --- | --- | --- | --- | --- | --- |
| A | 95.89 | - | - | - | - | - | 2.44 | 0.08 | 1.59 |
| B | 75.55 | - | - | - | - | - | 18.74 | 0.12 | 5.59 |
| C | 79.98 | - | - | - | - | - | 9.51 | 7.24 | 3.27 |
| D | 89.61 | 5.18 | 3.05 | - | - | - | 2.16 | 0.06 | 1.84 |
| E | 64.92 | 4.94 | 2.69 | - | - | - | 20.51 | 0.10 | 6.84 |
| F | 72.79 | 4.76 | 2.84 | - | - | - | 8.62 | 6.19 | 4.80 |
| G | 78.26 | - | - | 7.24 | 8.13 | 2.18 | 3.09 | 0.05 | 1.05 |
| H | 73.36 | - | - | 6.94 | 8.07 | 2.05 | 19.70 | - | 6.14 |
| I | 64.31 | - | - | 6.55 | 7.88 | 2.15 | 7.38 | 6.53 | 5.20 |

| Simulated conditions | Reagents and amounts | | | | | pH | Conductivity (mS·cm^-1^) |
| --- | --- | --- | --- | --- | --- | --- | --- |
|  | PBS | H_2_O_2_ | HCl | BSA | CLH |  |  |
| Normal | 5 standard tablets in 1 L deionized water | - | - | - | - | 7.4±0.1 | 15.7±0.5 |
| Inflammatory |  | 150 mM | 50 mM | - | - | 5.2±0.1 | 19.4±0.3 |
| Severe inflammatory |  |  |  | 10 g·L^-1^ | 10 g·L^-1^ | 3.0±0.2 | 16.1±0.1 |

Supplementary Table ST6. Identification names for the simulated solutions with their respective composition, pH and conductivity at 37 °C.
